# A Biopsychosocial Intervention for Stroke Carers (BISC): development and description of the intervention

**DOI:** 10.1080/21642850.2021.2016412

**Published:** 2022-01-02

**Authors:** Eirini Kontou, Shirley A. Thomas, Christine Cobley, Rebecca Fisher, Miriam R. Golding-Day, Marion F. Walker

**Affiliations:** a School of Medicine, University of Nottingham, Medical School, Queen’s Medical Centre, Nottingham, UK; b Institute of Mental Health, Nottinghamshire Healthcare NHS Foundation Trust, Nottingham, UK; c Department of Clinical Psychology, Derbyshire Healthcare NHS Foundation Trust, Derby, UK

**Keywords:** Stroke, carers, biopsychosocial, intervention development, group intervention

## Abstract

**Objective:**

Family members of stroke survivors are often not supported for their caring role, with many reporting adjustment difficulties. This paper describes the development and content of a group-based intervention for informal carers of stroke survivors.

**Method:**

The intervention is based on the theoretical foundation of the biopsychosocial model with the aim to understand and address the physical, psychological and social factors of caring for stroke survivors. Findings from a comprehensive literature review and a qualitative study with carers and stroke professionals were synthesized to guide the intervention development. The Template for Intervention Description and Replication (TIDieR) checklist was used as a framework to describe the intervention.

**Results:**

The intervention integrates cognitive-behavioural approaches via the identification of the biopsychosocial (physical, emotional, social) factors that can have an impact on the well-being of carers. It includes education on stroke-specific topics and advice on coping strategies. It consists of six structured two-hour group sessions facilitated in a community setting. It provides information and support on adjusting to the caring role in the first year post-stroke. Intervention materials were designed for addressing carers’ specific needs using psychological techniques, such as problem-solving, goal setting and relaxation exercises.

**Conclusion:**

We have underlined the importance for describing and reporting the process of intervention development for complex interventions in the context of stroke rehabilitation. An intervention addressing the needs of informal stroke carers (Biopsychosocial Intervention for Stroke Carers; BISC) has been developed and described. BISC was further evaluated in a single-centre feasibility randomized controlled trial.

Trial registration number ISRCTN15643456; Completed.

## Introduction

Stroke can have devastating consequences, and caring long-term for a stroke survivor can have a variety of adverse effects on the entire family (Stroke Association, 2019). Informal carers, often spouses or family members, play an important role in the early rehabilitation and long-term management of stroke survivors (McCullagh, Brigstocke, Donaldson, & Kalra, 2005). Carers of stroke survivors are more commonly female than male spouses (Kniepmann, 2012) with gender differences observed in the adjustment process to the caring role (Alexander & Wilz, 2010). Several studies report greater physical and emotional wellbeing among female carers of stroke survivors (Kniepmann, 2012). A systematic review reported a 25–54% prevalence of carer burden for an indefinite period following stroke (Rigby, Gubitz, & Phillips, 2009). The UK’s national stroke charity conducted a national survey and found that 85% of carers don’t get the support or information they need (Stroke Association, 2019). Family members providing informal and unpaid care are referred to as carers throughout this paper.

Little evidence is available on the effectiveness of interventions targeted at carers and the provision of carer-specific services can be limited (Victor, 2009). Research has identified the importance of education and support tailored to the specific and varied needs of stroke carers across the care pathway (Cameron & Gignac, 2008). However, only a minority of interventions have been developed, evaluated and implemented specifically for carers of stroke survivors (Robinson et al., 2005) and evidence regarding effectiveness is uncertain and limited (Cheng, Chair, & Chau, 2014; Forster et al., 2012; Legg et al., 2011).

One review (Legg et al., 2011) identified a structured stroke caregiver training programme that demonstrated improved outcomes in a single-centre feasibility Randomised Controlled Trial (RCT) (Kalra et al., 2004). In a subsequent pragmatic multicentre trial, the intervention was found to have no effect on carers’ burden when delivered by multidisciplinary hospital stroke teams (Forster et al., 2013).

Recently, many interventions aimed at supporting carers of stroke survivors have been evaluated with some preliminary positive results (Araújo, Lage, Cabrita, & Teixeira, 2018; Day et al., 2021) and others reported more conclusive findings but with no evidence on clinical or cost-effectiveness (Patchwood et al., 2021). However, many of these interventions often neglect to address the physical and psychological well-being of carers and focus more on the provision of information and practical support for preparing to manage the complex needs of stroke survivors for the transition from hospital to home care. In the UK and India, this involved multidisciplinary intervention studies for training carers to support stroke survivors with personal care, activities of daily living or other rehabilitation tasks (Forster et al., 2014; Lindley et al., 2017).

A systematic review of psychological health interventions for carers of stroke survivors underlined the importance of such interventions for reducing psychological distress and highlighted that interventions tailored to meet carers’ specific needs showed more favourable outcome (Panzeri, Rossi Ferrario, & Vidotto, 2019).

We have developed the Biopsychosocial Intervention for Stroke Carers (BISC) which is a new bespoke intervention specifically targeted to the needs of carers after discharge from hospital and within the first year post-stroke. The intervention was designed based on the priorities identified by key stakeholders and by following recommendations for the development of complex interventions (MRC, 2006). The acceptability and feasibility of this intervention compared to usual care were evaluated in a UK-based single-centre feasibility RCT (Trial registration: ISRCTN15643456) and the trial protocol has been described (Walker, Thomas, et al., 2017). The feasibility trial results were published in a subsequent paper with the primary focus reporting rates of recruitment, retention of participants in the intervention and completion of follow-up outcome measures (Walker et al., 2020). Findings suggested that the intervention content was acceptable and informative, however, the feasibility paper did not describe the process of intervention development or provide a detailed description of the intervention. The feasibility trial reinforced the importance of reporting the process and robustness of intervention development before considering further evaluation in a future definitive RCT. Few studies in stroke rehabilitation clearly or adequately describe how complex interventions are developed thereby hindering their use and application in clinical and research practice (Walker, Hoffmann, et al., 2017). Describing the intervention development process and detailed content of a new complex intervention has the potential to facilitate learning about future intervention research (Duncan et al., 2020).

This paper aims to describe the process of developing the BISC intervention for carers of stroke survivors and to supply a detailed description of the intervention elements ahead of a further evaluation.

## Intervention development

**Figure 1.**
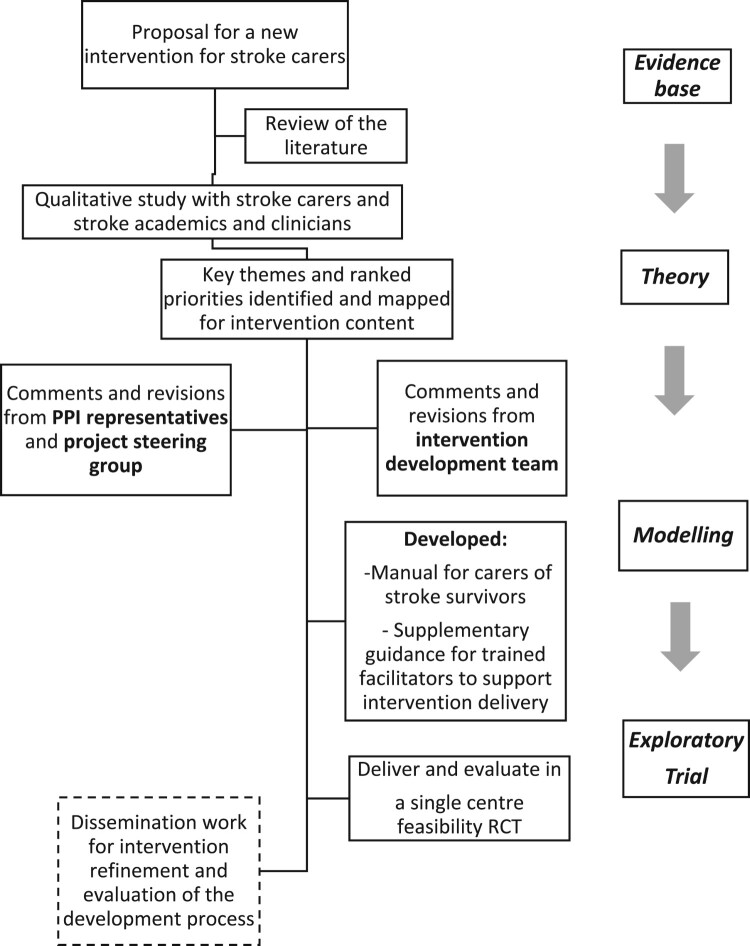
Flowchart for the development process of an intervention for stroke carers.

The intervention development was an integral part of a mixed-methods project with two distinct phases: a qualitative study (Condon, Benford, Kontou, Thomas, & Walker, 2019) and a feasibility RCT with a nested qualitative study (Walker et al., 2020; Walker, Thomas, et al., 2017). This overall research project received favourable ethical approval from the National Health Service Research Authority (ref: 14/EMI/1264). Particular attention was given to the key stages of intervention development recommended by the Medical Research Council framework (Campbell et al., 2000; MRC, 2006). The TIDieR guidelines and the consensus-based core recommendations for the development of stroke rehabilitation interventions (Hoffmann et al., 2014; Walker, Hoffmann, et al., 2017). Figure 1 describes the overall process of intervention development.

The intervention was developed by incorporating the findings from a literature review and a qualitative study to establish priority areas. The qualitative study involved focus groups and individual interviews with carers of stroke survivors and a nominal group technique discussion with stroke care academics and clinicians. A final consensus on the shared priority topics for the intervention was agreed; this was presented in a separate published paper (Condon et al., 2019).

The intervention manual was designed and reviewed by a multidisciplinary group of stroke researchers, professionals and Patient and Public Involvement (PPI) representatives. This ensured that relevant knowledge and experience among the study team was used to develop an appropriate intervention manual that would be relevant and acceptable for applying the intervention in a single-centre feasibility RCT (Walker et al., 2020).

## The BISC intervention (‘Biopsychosocial Intervention for Stroke Carers’)

The theoretical underpinning of the intervention is based on the biopsychosocial model of health, which is a framework that integrates the biological, psychological and social factors that are known to influence an individual’s well-being (Engel, 1977).

This model is now widely used in research investigating complex healthcare interventions and provides a holistic account of health and illness behaviours whilst focused on the person and their contexts (Wade & Halligan, 2017). It takes into account several factors that may affect an individual’s presenting difficulties in the context of rehabilitation (Wade, 2015) and therefore can be relevant for the understanding of the impact of stroke on carers’ health (physical and emotional).

The BISC intervention combines education about the biological, psychological and social effects of stroke and ways of adapting to the caregiving role. It aims to equip carers of stroke survivors with proactive coping strategies focussing on adjusting to their new role. The intervention provides information, advice and support on different topics, while highlighting the importance of self-care and setting achievable goals while caring for a family member or friend.

This paper presents a description and structure of the BISC intervention in accordance with the TIDieR checklist for better reporting of interventions (Hoffmann et al., 2014) (Table 1).

**Table 1.** Characteristics of the Biopsychosocial Intervention for Stroke Carers (BISC).

Description of the BISC intervention based on the TiDIER checklist (**Hoffmann et al., 2014**)	
Lay title	*‘Looking after your health and well-being when caring for someone who has had a stroke’*
Participants	Informal carers within a year post-stroke onset willing to attend a group intervention and to provide consent. Able to communicate in English with no visual/auditory impairments that would preclude them from participating in the sessions
Facilitator	Assistant psychologist or professional with equivalent skills and competencies with background knowledge in psychology. Some experience in basic cognitive-behavioural therapy principles is recommended. Training and clinical supervision are required
Duration	Six structured two-hour sessions once weekly. Depending on group size
Method of delivery	Group format for up to five participants
Setting	Accessible location in the community
Materials	Intervention manual for participants including home practice worksheets and session summaries. Supplementary notes to guide intervention delivery for the facilitator
Tailoring/modifications	The facilitator to follow the content and structure of the intervention manual. Some flexibility in the duration of group tasks/discussions and optional relaxation practice at the end of each session. If delivered on a one-to-one basis then modified to suit individual format

### Participants

The intervention is designed to be offered to informal carers (spouses, family members or friends) providing support for a stroke survivor who would have otherwise experienced considerable difficulties managing without their assistance and support after hospital discharge. The intervention is delivered to carers only. Stroke carers can opt in to take part in the intervention from the early stages of diagnosis and within the first year post-stroke.

### Materials

The intervention manual has the lay title ‘Looking after your health and well-being when caring for someone who has had a stroke’.

Each group session is focused on a particular topic, and content is presented flexibly using presentation slides, flipcharts or session handouts. The facilitator decides what format is most suitable according to group size and how interaction between group members unfolds.

The intervention manual containing the materials of all individual sessions is provided to carers at the first session. An extended version of this manual with session-by-session supplementary guidance is available for the facilitator to ensure that the content of the intervention is delivered consistently. Each session’s outline and summary are presented in the Supplementary Material; *BISC Manual Outline*.

### Facilitator and location

A facilitator with knowledge and understanding of stroke should deliver this intervention. For instance, it is intended that the sessions can be facilitated by an assistant psychologist or someone with the equivalent experience and expertise. The facilitator should receive specific training in the delivery of this intervention and clinical supervision by a healthcare professional with experience in stroke and/or mental health.

It is recommended that the intervention takes place in an easily accessible community setting after the stroke survivor has been discharged from the hospital and up to 1-year post-stroke. It is primarily designed to be delivered in a group format. Each group should ideally comprise up to five stroke carers. This group size allows more time to focus on each member’s experiences of caring for a stroke survivor and to ensure that the facilitator can effectively manage interactions between group members. Although this intervention is intended for delivery in a group format, it has the potential to be tailored by the facilitator if required to be offered on a one-to-one basis.

### Description of the intervention

The intervention consists of six structured two-hour group sessions focused on adjustment to stroke and the caregiving role, provision of education and psychological support. If participants are not able to attend a session, then the facilitator is available to debrief them and respond to any queries at the beginning of the next session.

Elements of the intervention draw upon cognitive-behavioural therapy principles, such as goal-setting and in-between session tasks. At the beginning of the intervention, the value of relaxation is introduced and an optional relaxation exercise is presented at the end of each session. Each session includes group discussions and an in-between session task (referred to as ‘Home Practice’). All sessions start with the participants reviewing progress with their ‘Home Practice’. This promotes engagement with the material learnt during the group sessions and setting achievable goals for the future.

The first session begins with an introduction about stroke and its effects on the survivor and carers. The biopsychosocial model is presented as a way of understanding the influence of different factors and how their interaction may affect an individual’s well-being. Participants are introduced to the topics addressed as part of this intervention and they are given time to get to know each other. During the second session, education is provided about adjusting to the effects of stroke and the caregiver role. Sessions 3, 4, 5 introduce cognitive-behavioural therapy (CBT) principles for noticing difficult emotions and managing unhelpful thoughts, dealing with problems and identifying strategies for coping more effectively. Session 6, the final session, summarises all previous sessions and starts with the idea of creating a well-being action plan (referred to as a ‘Wellbeing Toolbox’). This task has a practical focus and it aims to help stroke carers reflect on what they have learned and what strategies they can best implement to cope with present or future setbacks.

An overview of the intervention sessions with specific examples for each session is shown in Table 2.

**Table 2.** Overview of intervention sessions.

Sessions	Content	Techniques	Home practice
1. Stroke and caring	What is a stroke and its effects, caregiving role, biopsychosocial model of well-being	Introductions and getting to know each other ice-breaker task	Record of thoughts-emotions-behaviours during a caring situation
2. Adjustment and mood	Process of adjustment and normalisation	Discuss stages of adjustment	Identify what helps/gets in the way of adjusting
3. Emotions and thoughts	Dealing with difficult feelings and thoughts	Identify current and alternative strategies	Mood and activity diary
4. Dealing with problems	Learning problem-solving skills	Looking at the pros and cons when choosing a solution	Work through a particular problem
5. Stress and coping	Signs and causes of stress, coping strategies	Use the ‘stress barrel’ analogy	Try out a coping strategy identified as helpful
6. Your wellbeing action plan	Signposting and setbacks	Use ‘well-being toolbox’ analogy	Develop a personal well-being action plan

## Discussion

Given the urgent need to support carers for stroke survivors (Stroke Association, 2019), we developed a new group-based intervention, named BISC, for promoting the well-being of people who take on an informal caring role.

The phase of intervention development is often under-reported or not adequately described when published as part of a feasibility or pilot study (Duncan et al., 2020). This paper aims to describe the iterative process of developing a complex intervention by incorporating evidence from theory, research and collaboration with key stakeholders for determining its content for carers of stroke survivors.

The overall aim of the BISC intervention was to provide carers with the education and practical tools needed to adjust to the physical, psychological and social stresses that they may experience taking on the caring role within the first year post-stroke (Hall, Crocker, Clarke, & Forster, 2019). To achieve this goal, we developed an intervention manual and materials based on the biopsychosocial model of wellbeing (Engel, 1977), including sessions focused on the effects of stroke, adjustment to the caregiving role and coping strategies to look after their wellbeing. Instead of focusing on problems, the intervention is shaped by ideas and discussions on generating solutions, setting goals and developing plans for future setbacks in therapeutic progress. The effectiveness of psychological techniques based on cognitive-behavioural and problem-solving therapy techniques has been reported in other intervention studies for improving the psychological health of stroke carers (Panzeri et al., 2019).

A strength of this study is that the topics covered during all group sessions of the BISC intervention emerged from a review of the relevant literature, interviews with stroke carers and a nominal group consensus meeting with expert clinicians and researchers (Condon et al., 2019). Another key element of the intervention development process was patient and public involvement (PPI) and such a collaborative approach is recommended in intervention studies organising support for carers of stroke survivors (Patchwood et al., 2021). This allowed the tailoring of the intervention to the specific needs of the people providing care to stroke survivors as focused interventions have been found to be more effective and efficient (Panzeri et al., 2019). Involving carers in the development process ensured that the intervention is acceptable and developed in ‘real’ partnership with a multidisciplinary team of stroke professionals (Brereton, Carroll, & Barnston, 2007).

Another strength is that the proposed intervention has been manualised, with written materials available for the participants and additional notes for the facilitator. This is essential for research purposes, but also for future implementation of evidence-based interventions in clinical practice with documented advantages about intervention efficacy as well as the training and supervision of facilitators (Wilson, 1998).

The BISC intervention is designed to be delivered by an assistant psychologist or a healthcare professional with skills and competences in basic psychological principles. This may be considered a strength as a recent systematic review suggested that interventions for stroke carers led by psychologists showed more positive outcomes (Panzeri et al., 2019). It also enables other stroke professionals such as occupational therapists, rehabilitation assistants or nurses to be trained to deliver this intervention with appropriate support and supervision by a qualified psychologist.

As the intervention was designed to be delivered to carers early post-hospital discharge in the stroke care pathway, it could be regarded as preventative against worsening psychosocial difficulties. It is primarily designed as a low-level intervention providing education, self-help and signposting. This is in line with the provision of stepped psychological care after stroke, and the need for providing interventions for stroke survivors and their families adjusting to life after stroke (National Improvement Programme, 2011). However, this may be considered a limitation since it reduces its generalizability for use with carers who may suffer from depression or other significant mental health difficulties.

The different phases of intervention development (e.g. establishing intervention priorities, stakeholder involvement) do not necessarily mean that all uncertainties have been addressed. For example, we were not able to specify the criteria for the optimal delivery format (e.g. one-to-one versus group format) and this is yet to be determined. Although group-based interventions may be less acceptable and accessible to some people, there are several reasons why they may be more beneficial to others, such as peer support and social learning (Sadler, Sarre, Tinker, Bhalla, & McKevitt, 2017; Sörensen, Pinquart, & Duberstein, 2002). Delivering the intervention in a group format is also likely to be more time and cost-efficient (Söchting, 2014), which would be important given the high demand and limited provision of psychological input in stroke services (Sentinel Stroke National Audit Programme, 2020).

A wide range of approaches could have informed the development of this intervention which is underlined by cognitive-behavioural therapy principles. The intervention development group aimed to reach consensus on which strategies and techniques lend themselves well to a time-limited group intervention that aims to provide education and emotional support (Condon et al., 2019; Söchting, 2014). The involvement of individuals with research knowledge and clinical experience in stroke ensured that this intervention integrates multiple interacting components and has a multidisciplinary focus.

The Medical Research Council Framework (MRC, 2006; Skivington et al., 2021) for the development and evaluation of complex interventions identifies intervention development as the first of a series of interconnected phases. However, to our knowledge there are very few published intervention development studies aimed at supporting carers of stroke survivors (Hall et al., 2019). It is important to acknowledge that there are many ways to develop a new complex intervention, however, the success and appropriateness of the intervention development process can be evaluated in line with the consensus-based GUIDED recommendations (Duncan et al., 2020).

This paper highlights the iterative process of developing the BISC intervention development and provides an example for reporting intervention development research which can guide other researchers looking at investigating the effectiveness of supportive interventions for carers of stroke survivors. Clinicians could potentially identify some intervention components that can be incorporated in their everyday practice when supporting carers of stroke survivors, such as the consideration of the biopsychosocial model of health and wellbeing (Wade & Halligan, 2017).

The intervention is not yet suitable for implementation in clinical practice as its efficacy has yet to be determined. Further information as to how this intervention might be further refined and evaluated has been described in the BISC feasibility randomised controlled trial results paper (Walker et al., 2020). Future work is required to address any uncertainties regarding the intensity, mode of delivery, materials, or type of location that the intervention is most suitable for. The current paper describes the BISC intervention and can inform future research to optimise support interventions for carers of stroke survivors.

## Key messages

- Carers of stroke survivors can develop psychosocial difficulties related to their adopted caring role.
- A group-based intervention was co-developed from priorities identified by a multidisciplinary group of stroke carers, clinical and academic stroke professionals.
- The BISC intervention aims to provide education and support for carers in the first year after stroke. This paper describes the content and delivery of the BISC group-based intervention, which was further evaluated in a feasibility randomised controlled trial.
- Describing the process of intervention development can enable lessons to be learnt and incorporated into future intervention studies aimed at improving the well-being of carers.
- Future work is required to enhance the development and reporting of complex interventions for supporting carers of stroke survivors.

## Authors’ contributions

EK, SAT, MFW, MGD drafted the manuscript. MFW, CC, RF and SAT conceived the study and are grant holders. MFW is the principal investigator. MFW, EK and SAT contributed to the conception and development of the intervention, all authors commented critically on the manuscript, read and approved the final manuscript.

## Data Availability

The authors confirm that the data supporting the findings of this study are available within the article and its supplementary materials.
